# Investigating the Effects of Level-Specific CE-Chirp on Auditory Brainstem Response Waves in Normal Hearing Infants

**DOI:** 10.21315/mjms2024.31.2.7

**Published:** 2024-04-23

**Authors:** Norashikin Chahed, Ahmad Aidil Arafat Dzulkarnain, Saiful Adli Jamaluddin

**Affiliations:** 1 Department of Audiology and Speech-Language Pathology, Kulliyyah of Allied Health Sciences, International Islamic University Malaysia, Pahang, Malaysia; 2 Audiology Unit, Otorhinolaryngology Department, Hospital Ampang, Ministry of Health of Malaysia, Malaysia

**Keywords:** auditory brainstem response, brain stem, evoked potential, infants, LS CE-Chirp

## Abstract

**Background:**

Auditory brainstem response (ABR) to the level-specific (LS) CE-Chirp has been reported to provide optimum neural synchrony along cochlear partitions, theoretically improving ABR waveform resolution. Despite this promising finding, limited studies have been conducted to contrast the results between LS CE-Chirp and Click stimuli. The current study aimed to compare the results of ABR between the two stimuli (Click and LS CE-Chirp).

**Method:**

Sixty-seven normal-hearing infants, both with and without risk factors, aged less than 7 months old, participated in this study. The ABR test was conducted at 70 dBnHL using 33.3 stimulus repetition rates with both Click and LS CE-Chirp stimuli. The signal averaging was stopped at a maximum fixed signal average of 2,500 sweeps. Data were statistically compared between the two stimuli using the Wilcoxon signed-rank test.

**Results:**

The waves I and V ABRs elicited by LS CE-Chirp exhibited significantly larger amplitudes than the Click stimulus. However, the amplitude of wave III and absolute latencies were similar in both stimuli at a supra-threshold level.

**Conclusion:**

LS CE-Chirp has the advantage of larger amplitudes than the ABR from Click at the supra-threshold level (70 dBnHL) in normal-hearing infants.

## Introduction

Since the emergence of auditory brainstem response (ABR), non-level-dependent stimuli such as Click, tone burst and upward Chirp have been used to elicit ABRs until the development of the level-specific CE-Chirp (LS CE-Chirp) (1–3). LS CE-Chirp is the latest innovative stimulus with at least two advantages compared to other stimuli: i) it can compensate for the broader excitation at a high-intensity level in Chirp, and ii) it overcomes the ‘travelling wave delay’ issue in the Click stimulus.

The ‘broad excitation at the high-intensity level and changes in the cochlear-neural delay at the low-intensity level’ refers to a situation in which, at a high stimulus level, the excitation of cochlear regions broadens but narrows at a low stimulus level (4). The theory underlying the development of LS CE-Chirp is the delay in stimulus presentation and compensation concept. Therefore, following the specified delay model, a shorter stimulus presentation at a high-intensity level is suggested compared to the mid and low-intensity levels (2). This allows LS CE-Chirp to reduce neural firing desynchronisation and compensate for the negative effect of broader arousal in Chirp stimuli (2, 5, 6).

Chirp stimuli can overcome the ‘travelling wave delay’ in the Click stimulus. The original idea of the Chirp stimulus was based on the works of Shore and Nuttall (6), who introduced a rising frequency Chirp based on a model from the tone burst stimulus. Later, Dau et al. (1) developed a Chirp using the travelling wave delay approach from de Boer’s (7) cochlear linear model and Shore and Nuttall’s (6) original works. The Chirp stimulus organises the time-frequency function based on the tonotopic organisation of the cochlea. Specifically, the low-frequency component is presented first as it requires a long travelling distance in the cochlea, followed by mid and eventually high frequencies to activate the entire basal membrane simultaneously (1). This approach results in greater neural firing synchrony, leading to a larger wave V amplitude in the Chirp than the ABR to Click stimulus (8).

The Click stimulus is a traditional stimulus used for over 5 decades to elicit synchronous firing of auditory neurons. Due to the cochlea’s tonotopic organisation, the Click stimulus’s low and higher-frequency components do not simultaneously arrive at the cochlea for activation. This results in ABR neuronal activities capturing responses mainly from the basal turn, while the responses from the apical region of the low-frequency region are out of phase. This can lead to missing responses from individuals with low-frequency hearing loss (9).

A study investigating LS CE-Chirp ABR with different stimulus polarities reported that LS CE-Chirp produces larger ABR amplitude of wave V than the Click stimulus in all stimulus polarities (10). This finding is consistent with other studies that reported greater ABR amplitudes of waves I, III and V with LS CE-Chirp compared to the Click stimulus in adults (5, 11–14) and infants (15). Xu et al. (16) further supported this notion by reporting that the larger amplitude resulting from LS CE-Chirp led to a hearing threshold estimation close to the behavioural audiogram (within 3 dBHL–5 dBHL) in hearing-impaired young children. ABR to CE-Chirp and LS CE-Chirp findings were similar to adult behavioural audiograms from low to mid frequencies (17–19).

Despite many previous studies reporting the advantages of LS CE-Chirp over other stimuli in eliciting ABR, most investigations have focused on different population groups, such as young children (16) and adult populations (5, 11–13, 20). To our knowledge, only one study has investigated LS CE-Chirp in 18 non-risk infants (15). This study revealed higher ABR amplitude and delayed latencies with LS CE-Chirp than Click stimuli in certain combinations. However, this recent study had a small sample size and the authors did not include the analysis of wave I and III amplitudes, which are important measures, especially for LS CE-Chirp stimuli. LS CE-Chirp can minimise the upward spread of excitation at high-intensity levels, stimulating the slow fibres of both wave I and III, unlike the previous version of the broadband Chirp stimulus.

As most studies concerning ABR with LS CE-Chirp were conducted in adults, the results reported in the adult population may differ from those in infants due to the ongoing maturational process of the central auditory system in infants (21–23). For instance, the latency and interpeak latency (IPL) are delayed in newborns compared to adults (24). Additionally, previous findings from other types of infant stimuli cannot be generalised to LS CE-Chirp since Click stimuli have less neural synchronisation in low frequency. Cochlear regions and most findings from the stimulus represented neural responses between 2 and 4 kHz (25, 26). Moreover, the traditional Chirp stimulus is less efficient at higher stimulus intensity levels, resulting in the absence of earlier ABR waves I and III compared to the CE-Chirp and Click stimuli (3, 27).

However, previous studies have reported larger ABR amplitude and improvements in waveform resolution when elicited by LS CE-Chirp and Chirp stimuli in adults and infants. This allows for easy waveform detection even at low-intensity levels, lowering the ABR hearing threshold estimation level (9, 19). Considering these factors, further investigations are warranted. The current research compared LS CE-Chirp and Click ABR amplitude and latency (waves I, III and V).

## Methods

### Study Participants

The study participants, including 67 normal-hearing infants, with a mean ± SD age of 3.75 ± 1.96 months old. Only one ear was randomly tested for each infant, analysing 32 ABRs from the right ear and 35 from the left ear. Among the participants, 55 infants had high-risk factors, while 12 infants were born without any high-risk factors. Table 1 summarises the high-risk factors associated with the 55 infants, with neonatal jaundice (NNJ) being the majority risk factor.

All infants were recruited from the newborn hearing screening (NHS) programme under the Audiology Unit of Sultan Ahmad Shah Medical Centre (SASMEC) in Kuantan, Pahang and Hospital Ampang in Selangor. The recruitment process involved conducting a distortion product of otoacoustic emission (DPOAE) screening test within 8 h to 24 h post-delivery. Infants who passed the DPOAE screening were invited to join the study, while those who failed were scheduled for automated auditory brainstem response (AABR) as a second screening within 4 weeks. Upon obtaining parental consent, the ABR appointment date was scheduled.

The participants met the preliminary study criteria, which included: i) normal hearing based on the DPOAE or AABR screening results, ii) no notable family history of hearing loss, iii) clear external auditory canal and intact tympanic membrane as observed during the otoscopic examination, iv) type A tympanogram indicating normal middle ear function based on high-frequency tympanometry test, v) no significant medical history and normal body temperature (36.8 °C–37.5 °C) and vi) baseline ABR to Click stimulus showing normal hearing estimation level. The ABR test was continued with LS CE-Chirp stimulus in the main study for infants with a normal hearing threshold estimation level.

### Procedure

A two-channel interacoustics eclipse EP15 ABR system was used at two centres: i) the electrophysiology room of IIUM Hearing and Speech Clinic in Kuantan, Pahang and ii) the audiology room at Hospital Ampang in Selangor. Once the infants were in a sleep state, their skin was prepared by applying NuPrep^®^ gel on four specific areas: i) high forehead (Fz), ii) low forehead (Fpz) and mastoid areas on both ears (iii) M1 and iv) M2. The Sanibel^™^ snap electrodes were connected to the Auditory Evoked Potential (AEP) system and placed in the prepared areas.

After the skin preparation, the impedance of each electrode was measured. The impedance of all individual electrodes was less than 5 kΩ and the impedance between electrodes was less than 2 kΩ. A proper seal was ensured by inserting a 3.5 mm or 4.0 mm Eclipse infant insert ear tip or a pediatric 3B 3.5 mm ear tip. The recording montage used ipsilateral and contralateral recordings, with the non-inverting electrode placed on the Fz, the ground electrode on the Fpz and the inverting electrodes on both mastoid areas (M1 and M2). A ± 15 μV artefact rejection level, a bandpass filter (33 Hz–3,000 Hz) and a 14 ms recording epoch were used. Bayesian-weighted averaging was employed in this study.

A monoaural ABR test was conducted on infants during sleep, either in a natural sleep state or through chloralhydrate sedation. The main study involved an ABR test using the alternating polarity of Click and LS CE-Chirp stimuli, with both spectra ranging from 200 to 11,000 Hz at 33.3 cps and presented at 70 dBnHL. A 30 dBnHL white noise masker was presented at the contralateral ear to prevent crossover or contribution from the non-test ear. The stimuli were calibrated by the manufacturer using reference threshold levels from the Interacoustic Standard test Signal International Electrotechnical Commission (IEC) 60645-3 (2007) and AEP IEC 60645-7 (2009) Type 1. The reference threshold level for air-conducted ABR at 100 dB was evaluated using a sound level meter (Brüel & Kjær ½” Type 2250) and a free-field microphone (Brüel & Kjær ½” type 4191). The 0 dBnHL equivalency to the dBpeSPL was set at 35.5 dBpeSPL for LS CE-Chirp and 31.5 dBpeSPL for Click stimulus. The ABR signals were averaged over 2,500 sweeps and only ABRs with a minimum signal-to-noise ratio (SNR) of 3.0 were accepted.

### Waveform Analysis

The amplitude was determined by measuring the voltage change (μV) between the peak and trough following waves I, III and V. Latency was defined as the time taken for the respective wave to reach a positive peak after the stimulus presentation, measured in milliseconds (28). The presence of ABR waveform components was determined by an experienced audiologist with 13 years of experience. Before the actual ABR interpretation, the audiologist and a second audiologist with 18 years of experience independently interpreted a few selected samples of the ABR waveforms from the study. Their interpretations were compared and any discrepancies were discussed. This process was repeated for a few sessions until an inter-rater agreement of approximately 90% was reached. Subsequently, the first audiologist independently interpreted the ABR findings.

### Data Analysis

The main research variables were the ABR amplitude and latency (waves I, III, and V) at 70 dBnHL. Based on the Kolmogorov-Smirnov test, most data were not normally distributed (*P* < 0.05). Therefore, non-parametric tests were chosen, including the Friedman and Wilcoxon signed-rank tests at a 95% confidence level. The Friedman test was used to compare the amplitudes and latencies of waves I, III and V for each stimulus, and the Wilcoxon signed-rank test was employed for post hoc comparisons as applicable. The Wilcoxon signed-rank test was also used to compare the ABR variables between the two stimuli. A *P*-value less than 0.01 was considered significant. The effect size of the Wilcoxon signed-rank test was determined by calculating the ratio of the statistic (*Z* score) divided by the square root of the sample size (*N*) (29).

## Results

**Figure 1:**
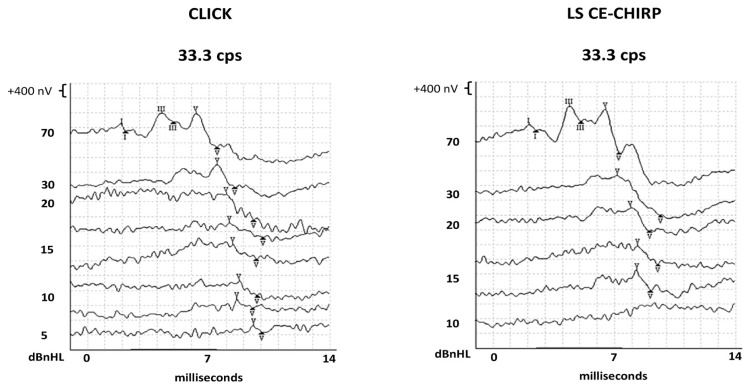
The ABR waveforms from Click and LS CE-Chirp stimuli at multiple intensities levels from one of the study participants

Figure 1 demonstrates the waveform of ABR elicited by Click and LS CE-Chirp stimuli at multiple intensity levels from one of the infants. Overall, waves I and V from all 67 infants were identified 100% in both stimuli ABRs. However, one participant’s wave III from the ABR to Click stimulus was not identified at 33.3 cps at 70 dBnHL (98.5% detection). In contrast, wave III was identified in all participants (100%) in the ABR to the LS CE-Chirp stimulus. More details are shown in Table 2.

### Comparison of Amplitudes (Waves I, III and V) between Stimulus Types

Table 3 summarises the median and interquartile range (IQR) of ABR amplitudes (waves I, III and V) elicited by Click and LS CE-Chirp stimuli at 33.3 cps and 70 dBnHL. For the Click stimulus, the Friedman test demonstrated a statistically significant difference in ABR amplitudes among all waves (I, III and V) (χ^2^ (2, *N* = 66) = 83.108, *P* < 0.001). Similarly, for LS CE-Chirp, the Friedman test showed a statistically significant difference in ABR amplitudes among all waves (I, III and V) (χ^2^ (2, *N* = 67) = 96.415, *P* < 0.001).

Post hoc analysis using the Wilcoxon signed-rank test and Table 3 revealed that the ABR amplitudes of waves I and V elicited by LS CE-Chirp were significantly larger than those elicited by the Click stimulus, with a medium to large effect size (wave I (*r* = 0.43): *Z* = −3.490, *P* < 0.001; wave V (*r* = 0.78): *Z* = −6.355, *P* < 0.001). No significant changes were observed in the ABR wave III amplitude between the two stimulus types, with a small effect size (*r* = 0.24) (*Z* = −1.971, *P* = 0.049). Further details of the *P*-values and effect sizes are presented in Table 4. The relative increment of ABR amplitudes from Click stimulus to LS CE-Chirp was 17% (IQR = 66.1) for wave I and 48% (IQR = 58.3) for wave V.

### Comparison of Absolute Latencies (Waves I, III and V) between Stimulus Types

Table 3 displays the median and interquartile range (IQR) of ABR absolute latencies (waves I, III and V) for LS CE-Chirp and Click stimuli. The Friedman test showed a statistically significant difference in absolute latencies among all waves (I, III and V) for both the Click stimulus (χ^2^ (2, *N* = 66) = 132.000, *P* < 0.001) and LS CE-Chirp (χ^2^ (2, *N* = 67) = 134.000, *P* < 0.001).

Post hoc analysis using the Wilcoxon signed-rank test and Table 3 revealed no significant changes in ABR absolute latencies for waves I, III and V between the two stimulus types (*P* > 0.01), with a small effect size (wave I (*r* = 0.10) (*Z* = −0.853, *P* = 0.393), wave III (*r* = 0.22) (*Z* = −1.797, *P* = 0.072) and wave V (*r* = 0.02) (*Z* = −0.201, *P* = 0.840)). Further details of the *P*-values and effect sizes are shown in Table 5.

## Discussion

The current research aimed to compare the ABR results (amplitudes and latencies of waves I, III and V) between two stimulus types: i) LS CE-Chirp and ii) Click. The results revealed larger ABR amplitudes (waves I and V) in LS CE-Chirp than the Click stimulus at a supra-threshold level in normal-hearing infants. These findings are consistent with earlier studies in adults that utilised the Click stimulus and LS CE-Chirp (5, 11, 13, 14) and comparative studies with tone burst stimuli (12). The larger amplitude observed in LS CE-Chirp can be attributed to its specific stimulus presentation sequence, which starts from low frequencies and progresses to high frequencies, thereby promoting neural synchrony across all regions of the basilar membrane (4, 30, 31). Increased neural synchrony leads to larger ABR amplitude in LS CE-Chirp due to the amount of synchronised neural activity (32). Additionally, LS CE-Chirp is designed to balance intensity level and stimulus duration with shorter stimulus presentation at higher intensity levels, thereby minimising the upward spread of excitation (2, 3, 5). Consequently, the efficiency in eliciting ABR responses and the increase in amplitude with LS CE-Chirp are observed at the supra-threshold level of 70 dBnHL (3, 5, 13).

In addition to amplitude, the current study examined ABR absolute latency in both stimuli. The absolute latencies of waves I, III and V were similar between the Click stimulus and LS CE-Chirp at the high-intensity level. This similarity suggests that the ABR system adjusts the onset and offset times of LS CE-Chirp for each frequency (33). During the construction of the CE-Chirp stimulus, the temporal sequence is adjusted by subtracting the value from the onset time to the offset time. As a result, the time for neural activities to reach the respective ABR peaks of I, III and V in LS CE-Chirp is comparable to the time in the Click stimulus. Technically, the present research findings indicate that the offset of LS CE-Chirp coincides with the onset of the Click stimulus (33, 34).

## Conclusion

The LS CE-Chirp shows promise as an effective method for determining ABR auditory threshold estimation in infants, comparable to the Click stimulus but with the advantage of larger amplitude. However, these conclusions are limited to normal-hearing infants (with and without risk factors) at a 70 dBnHL level and within the parameters used in the current study. The findings apply to infants who passed NHS and are less than 7 months old. Future studies could explore the applicability of these research findings to hearing-impaired infants and investigate the methodology and consistency of these findings in other populations.

## Figures and Tables

**Table 1:** The risk-factors of participants

		No. of infants
Non-risk		12
	NNJ	35
	Pneumonia	1
	Ototoxic medication	2
	TORCHES	2

High-risk factors	Syndromic	3
	Hypoxic ischaemic encephalopathy	–
	Cleft lip/palate	–
	More than one factor	12
	Total	67

**Table 2:** Number of subjects has presence ABR waveforms in each stimulus type at 33.3 cps

	Waves	I	III	V
	
Stimulus type	LS CE-Chirp	67	67	67
	Click	67	66	67

**Table 3:** The median, IQR and quartile for ABR amplitude and latency of waves (I, III and V) elicited by LS CE-Chirp and Click stimuli at 33.3 cps at 70 dBnHL

Stimulus intensity (dBnHL)	Waves	LS CE-Chirp		Click	
	
		Median (IQR)	Quartile 1; 3	Median (IQR)	Quartile 1; 3
Amplitude (μv)					
	I	0.21 (0.13)	0.16; 0.29	0.18 (0.10)	0.13; 0.23
	III	0.21 (0.11)	0.15; 0.26	0.18 (0.13)	0.12; 0.25
	V	0.59 (0.35)	0.42; 0.77	0.40 (0.20)	0.33; 0.53
Latency (ms)					
	I	2.25 (0.86)	1.83; 2.69	2.40 (0.57)	2.00; 2.57
	II	4.43 (0.31)	4.27; 4.58	4.37 (0.37)	4.20; 4.57
	V	6.47 (0.43)	6.20; 6.63	6.43 (0.44)	6.23; 6.67

**Table 4:** The *P*-values of the post hoc Wilcoxon signed-rank test and effect size (*r*) for the ABR amplitudes of waves I, III and V at 33.3 cps at 70 dBHL

Amplitude (μv)	Wave	*P*-value		Effect size (*r*)	
		
Stimulus		LS CE-Chirp	Click	LS CE-Chirp	Click
LS CE-Chirp	I				
Click		*0.000		*0.43	
LS CE-Chirp	III				
Click		0.049		0.24	
LS CE-Chirp	V				
Click		*0.000		*0.78	

Notes:

* Indicate significant *P*-value = *P* < 0.01 or more than medium effect size (*r* > 0.3)

**Table 5:** The *P*-values of the post hoc Wilcoxon signed-rank test and effect size (*r*) for the ABR absolute latency of waves I, III and V at 33.3 cps at 70 dBnHL

Absolute latency (ms)	Wave	*P*-value		Effect size (*r*)	
		
Stimulus		LS CE-Chirp	Click	LS CE-Chirp	Click
LS CE-Chirp	I				
Click		0.393		0.10	
LS CE-Chirp	III				
Click		0.072		0.22	
LS CE-Chirp	V				
Click		0.840		0.02	
